# Plastrum Testudinis Extracts Promote NSC Differentiation into Dopaminergic Neuron by Regulating the Interaction of TET1 and FoxA2

**DOI:** 10.1155/2020/9104135

**Published:** 2020-04-20

**Authors:** Jun Zhong, Sen Ye, Xiaoli Zhou, Jiapei Huang, Xican Li, Saixia Zhang, Jianhong Zhou, Dongfeng Chen, Caixia Li

**Affiliations:** ^1^School of Basic Medical Sciences, Guangzhou University of Chinese Medicine, Guangzhou, Guangdong Province, China; ^2^Research Center of Integrative Medicine, School of Basic Medical Sciences, Guangzhou University of Chinese Medicine, Guangzhou, Guangdong Province, China; ^3^NangFang College of Sun Yat-Sen University, Guangzhou, Guangdong Province, China; ^4^School of Pharmaceutical Sciences, Guangzhou University of Chinese Medicine, Guangzhou, Guangdong Province, China; ^5^The Center of Laboratory Animal, Guangzhou University of Chinese Medicine, Guangzhou, Guangdong Province, China

## Abstract

In recent years, stem cells have gained much attention for the treatment of neurodegenerative diseases. However, inducing neural stem cell directionally differentiation is a difficult problem in the treatment of Parkinson's disease (PD) by stem cell therapy. Plastrum Testudinis (PT) can enhance the number of TH-positive neurons in the PD rat brain substantia nigra, but the underlying mechanism has not been clarified. Here, we aimed at further investigating the mechanism by which PT can promote NSC differentiation into dopaminergic neurons. A rat model of PD was used for detecting the effect of PT on the rat brain substantia nigra in vivo. The results showed the expressions of tyrosine hydroxylase (TH) and TET1 enzyme were increased after treatment with PT. Consequently, Plastrum Testudinis extracts (PTEs) were used for inducing NSC differentiation into dopaminergic neurons ex vivo. During differentiation of NSCs induced by PTE, TH expression was increased, with a concomitant increase in both TET1 and FoxA2. Next, we performed coimmunoprecipitation analysis to examine the interaction between TET1 protein and FoxA2 protein. Our results show that PTE can increase the binding rate of TET1 and FoxA2. Thus, our findings show that PTE can increase the efficiency of NSCs to directionally differentiate into dopaminergic neurons and provide experimental evidence for PT in the treatment of Parkinson's disease.

## 1. Introduction

Parkinson's disease (PD) is a common progressive neurodegenerative disease that occurs in middle-aged and elderly people. Its main pathological change is degeneration and deletion of dopaminergic neurons in the substantia nigra, which leads to a series of clinical symptoms such as resting tremor, bradykinesia, muscle rigidity, and autonomic dysfunction. Because of the aging population, medical expenditure for PD (which is one of the diseases with the highest incidence of nervous system diseases) can be a considerable problem for the socioeconomic burden in the future [1]. Unfortunately, the pathogenesis of PD is still not clear. Until recently, levodopa was the main drug used for the treatment of PD. However, long-term use of levodopa is associated with the development of levodopa-induced motor complications [2]. Accordingly, some studies have suggested using cell replacement therapy as an alternative direction for the treatment of PD [3, 4].

Neural stem cells (NSCs) are the cell population of the nervous system with the potential for self-renewal and multidirectional differentiation, which could differentiate into neurons, astrocytes, and oligodendrocytes [5]. Researchers have shown that NSCs have good application prospects for the treatment of age-related neurodegenerative diseases [6]. Moreover, these studies have demonstrated that NSCs can differentiate into dopaminergic neurons and may reverse the degeneration process of PD by replacing the loss of dopaminergic neurons. Consequently, inducing directed differentiation of NSCs into dopaminergic neurons has become a key issue.

Studies have shown that DNA demethylation is indispensable for the differentiation of NSCs [7, 8]. Ten-eleven translocation 1 (TET1) is a member of the TET family that is highly expressed in embryonic stem cells and the nervous system and is an important enzyme in DNA demethylation [9]. When DNA demethylation occurs, TET1 converts 5-methylcytosine (5mC) to 5-hydroxymethylcytosine (5hmC) under the combined action of iron(II) and *α*-ketoglutarate acid-dependent dioxygenases [10]. Interestingly, 5hmC is a biomarker of DNA demethylation that is highly enriched in the brain and crucial during neural development [11]. Nevertheless, the mechanism by which DNA demethylation regulates NSC differentiation has not been clarified, yet many studies had revealed a relationship [12, 13]. Hahn et al. demonstrated that 5hmC levels were increased during neuronal differentiation in mouse brain and were associated with activation of genes important for related neuronal functions. Additionally, TET1 plays a key role in the transformation of human fibroblasts into induced dopaminergic neurons [14]. Recently, a study reported that liver progenitor cell differentiation requires TETs and forkhead box A2 (FoxA2), providing specific direction for our research [15]. The FoxA2 protein is a member of the forkhead family, with a wide range of expression in the midbrain. Further, FoxA2 is an essential nuclear transcription factor involved in the differentiation of NSCs into dopaminergic neurons [16]. One study demonstrated that deletion of FoxA2 causes a reduction in the number of TH-positive midbrain dopaminergic (mDA) neurons [17]. TH catalyzes the formation of L-dopa by tyrosine and is the rate-limiting enzyme of catecholamine synthesis. Further, TH has been used as a biomarker of dopaminergic neurons [18]. For all of the above reasons, we are interested in increasing the efficiency of the directional differentiation of NSCs into dopaminergic neurons.

Traditional Chinese medicine (TCM) can regulate the differentiation of NSCs by changing the microenvironment. Many studies have suggested that single TCM, extracts of TCM, and some TCM monomers can regulate differentiation of NSCs [19–21]. Plastrum Testudinis (PT; Carapax Testudinis, Guijia) is a TCM that has been shown to have potential in promoting osteogenic differentiation of bone marrow stem cells [22]. However, the mechanism by which PT induces NSC differentiation into dopaminergic neurons is not known precisely. Previous research from our group identified the active component of PT as ethyl acetate extracts from PT (i.e., PTE). In this study, we further investigated the mechanism by which PTE promotes NSC differentiation into dopaminergic neurons.

## 2. Experimental Procedure

### 2.1. Rat Model of Parkinson's Disease

This study was carried out in accordance with national animal experiment protocols and approved by the Experimental Animal Ethics Committee of Guangzhou University of Traditional Chinese Medicine (Protocol no. 20130035). All surgeries were performed under sodium pentobarbital anesthesia, and all efforts were made to minimize suffering.

The rat model was established by injecting 6-hydroxydopamine (6-OHDA) into the midbrain, according to the method used in a study [23]. Sprague Dawley (SD) male rats weighing 90–100 g were selected, anesthetized with 1% pentobarbital sodium, and fixed with a stereotaxic apparatus. The 6-OHDA was diluted in 0.9% NaCl solution. Taking the midpoint of the coronal suture as the origin, the height +7.8 mm, the ordinate −5.2 mm, and the abscissa +1.8 mm is the substantia nigra (SN); the height +7.8 mm, the ordinate −4.6 mm, and the abscissa +0.9 mm is the substantia nigra pars compacta (SNpc). The left SN and SNpc of rats were injected with 8 *μ*l 6-OHDA (4 *μ*g/*μ*l), respectively. And, at the two injection points in the sham operation group, the same volume of 0.9% NaCl solution was injected. One week after operation, rats were tested with apomorphine (0.5 mg/kg) by intraperitoneal injection once a week, and the number of rotations of the rats within 30 min was observed and recorded. The average rotational speed of more than 7 r/min was regarded as a successful PD rat model. The successful rat models were randomly divided into four groups: PD model group, positive drug group, low-dose drug group, and high-dose drug group, while the wild-type rats were randomly divided into the blank control group and the sham operation group. Each drug group was treated continuously for 45 days. The complete animal groupings and drug treatment measures are as follows: blank control group: normal SD rats without surgery, 1 ml/kg 0.9% NaCl solution per day by intragastric administration for each rat; sham operation group: the same volume of 0.9% NaCl solution was used instead of 6-OHDA during operation, and 1 ml/kg 0.9% NaCl solution per day by intragastric administration for each rat; PD model group: 1 ml/kg 0.9% NaCl solution per day by intragastric administration for each rat; positive drug group: 1 ml/kg positive drug solution per day by intragastric administration for each rat (10 mg/kg levodopa +2.5 mg/kg benserazide); low-dose drug group: 0.4 ml PT (1.1 g/d) per day by intragastric administration for each rat; and high-dose drug group: 3.4 ml PT (9.9 g/d) per day by intragastric administration for each rat.

### 2.2. Immunohistochemical Staining Assay

Rats were sacrificed by overinjecting 1% pentobarbital sodium (at least >150 mg/kg), and the brain tissues were fixed in 4% paraformaldehyde. Brain tissues were embedded in paraffin, and the tissues were sectioned to a thickness of 4 *μ*m. Staining of rat brain (substantia nigra) tissue sections was performed. The sections were deparaffinized by xylene and rehydrated by gradient ethanol solution (from 100% to 75%). Sections were inhibited for endogenous peroxidase activity using 3% H_2_O_2_ buffer at 37°C for 15 min. The antigen retrieval was done by using a microwave oven with citric acid solution (pH 6.0) at 100°C for 10 min, and then, the slices were allowed to cool sufficiently and equilibrated to room temperature. Sections were permeabilized by 0.5% Triton X-100 (diluted in PBS pH 7.4, PBS was purchased from Gibco, #C10010500BT) for 15 min at 37°C and blocked in 10% goat serum for 30 min at 37°C. Incubation with primary antibodies (polyclonal rabbit anti-tyrosine hydroxylase, 1 : 500, Abcam, # ab112; polyclonal rabbit anti-TET1, 1 : 100, Abcam, # ab191698; monoclonal rabbit anti-FoxA2, 1 : 200, Abcam, #ab108422; polyclonal rabbit anti-5-hydroxymethylcytosine, 1 : 200, Active Motif, # 39769) was performed overnight at 4°C and secondary antibody (biotinylated goat anti-mouse/rabbit IgG, Boster, #SA1020) was incubated at room temperature for 1 hour. Subsequently, streptavidin-biotin complex (SABC kit, Boster, #SA1020) was incubated at room temperature for 30 min, color development was observed by adding diaminobenzidine (DAB color development kit, Boster, #AR1022) for 10 min, and hematoxylin is used for counterstaining. The sections were imaged using Olympus image analysis system and analyzed using ImageJ software.

### 2.3. Preparation of Extracts from Plastrum Testudinis

PT and PTE were provided by Professor Xican Li, from the School of Pharmaceutical Sciences at Guangzhou University of Chinese Medicine. PTE was obtained based on the previously established methods [24, 25]. The part of ethyl acetate solvents was recovered and was dissolved in dimethyl sulphoxide (30 mg/mL).

### 2.4. Neural Stem Cells Isolated and Cultured

SD rats of E14-17 were provided from the Animal Center of Guangzhou University of Traditional Chinese Medicine. Neural stem cells were isolated from the embryonic brain of E14-17 SD rat according to a report [26]. Embryonic brain tissues were mechanically dissociated and digested by 0.25% tryptic-EDTA (Gibco). After digestion was completed, cells were cultured with serum-containing medium (DMEM/F12 1 : 1) supplemented with 10% fetal bovine serum to terminate the digestion and washed twice with phosphate-buffered saline (PBS). The single-cell suspension was prepared to use the blow of Pasteur pipette with based growth medium, which is a serum-free medium (DMEM/F12 1 : 1) supplemented with the growth factor Fibroblast Growth Factor-basic and Epidermal Growth Factor at 20 ng/ml (all from GenScript) as well as the 2% (v/v) B27,100 units/ml penicillin, and 100 *μ*g/ml streptomycin (all from Gibco). The cells were seeded into 75 ml culture flasks and cultured at 37°C in an environment of 5% CO_2_. The culture medium was changed every 3 days.

### 2.5. Induction and Differentiation of NSCs

When NSCs were at passage 2, single cell suspension was prepared by mechanical dissociation. Cells were plated into 6-well culture plates and cultured with serum-containing medium (10% FBS in DMEM/F12) supplemented with 100 units/ml penicillin and 100 *μ*g/ml streptomycin, and the PTE (3 *μ*g/ml, 30 *μ*g/ml) was immediately added to induce NSC differentiation. The culture medium was changed every 2 days. At 5 days of differentiation, TET1, FoxA2, global 5hmC levels, and TH expression were assessed.

### 2.6. Western Blot Analysis

Total proteins were lysed in RIPA lysis and extraction buffer (Thermo) with Protease Inhibitor Cocktail (EDTA-Free, 100X in DMSO (Bimake)) for 15 min on ice. Nuclear proteins were extracted by using Nuclear and Cytoplasmic Protein Extraction Kit (KeyGEN) according to the manufacturer's instructions. Protein concentration was quantified by BCA protein assay kit (FD) according to the manufacturer's instructions. The proteins were used for western blotting, separated by 10% SDS-PAGE (Beyotime), and then transferred to 0.45 *μ*m polyvinylidene fluoride (PVDF) membrane (Millipore). The membranes were blocked by 5% nonfat dry milk (Cell Signaling Technology) for 2 hours at room temperature. Primary antibodies were incubated overnight at 4°C, and secondary antibodies were incubated for 1 hour at room temperature, and the signals were detected by Immobilon Western Chemiluminescent HRP Substrate (Millipore). The following antibodies were used: rabbit anti-TET1 antibody (1 : 1000, polyclonal, Abcam, Cambridge, UK, no. ab191698), mouse anti-FoxA2 antibody (1 : 500, monoclonal, Abcam, Cambridge, UK, no. ab60721), rabbit anti-FoxA2 antibody (1 : 1000, monoclonal, Abcam, Cambridge, UK, no. ab108422), rabbit anti-tyrosine hydroxylase antibody (1 : 500, polyclonal, Abcam, Cambridge, UK, no. ab112), mouse anti-*β*-actin (1 : 500, monoclonal, Boster Biological Technology, Wuhan, CN, no. BM0627), rabbit anti-Lamin B1 (1 : 100, polyclonal, Boster Biological Technology, Wuhan, CN, no. PB0640), goat anti-rabbit IgG H&L (HRP) (1 : 10000, polyclonal, Abcam, Cambridge, UK, no. ab6721), and rabbit anti-mouse IgG H&L (HRP) (1 : 10000, polyclonal, Abcam, Cambridge, UK, no. ab6728).

### 2.7. Dot Blot Analysis

The genomic DNA samples were extracted by Takara MiniBEST Universal Genomic DNA Extraction Kit Ver.5.0 (Takara, Shiga, JP, no. 9765). The PVDF membrane was activated with methanol for 5 min. After the membrane dried, DNA samples were spotted onto the PVDF membrane, and then the membrane was left to dry at 60°C for 2 hours. The membranes were blocked by 5% bovine serum albumin for 2 hours at room temperature. The primary antibodies were incubated overnight at 4°C, and the secondary antibody was incubated for 2 hours at room temperature, and the signals were detected by Immobilon Western Chemiluminescent HRP Substrate (Millipore). The following antibodies were used: rabbit anti-5-hydroxymethylcytosine (1 : 10000, polyclonal, Active Motif, Carlsbad, USA, no. 39769) and goat anti-rabbit IgG H&L (HRP) (1 : 10000, polyclonal, Abcam, Cambridge, UK, no. ab6721).

### 2.8. Real-Time Quantitative PCR (qRT-PCR) Analysis

NSCs were plated into 6-well culture plates and cultured with the PTE (30 *μ*g/ml) for 5 days. Total RNA was extracted from different groups of cells by TRIzol reagent (Invitrogen) and Direct-zol™ RNA MiniPrep Kit (ZYMO) according to the manufacturer's instructions. About 2 *μ*g total RNA was reverse-transcribed into cDNA by PrimeScript™ RT reagent Kit (Takara, Shiga, JP, no. RR037A) according to the manufacturer's instructions, which is used for qRT-PCR. qRT-PCR was performed by using TB Green™ Premix Dimer Eraser™ kit (Takara, Shiga, JP, no. RR820A) and following the protocol: initial denaturation at 95°C for 30 sec, 40 cycles of PCR followed by 95°C for 5 sec, 58°C for 30 sec, and 72°C for 1 min, and the relative expressions of different genes were quantitatively analyzed by the 2^−ΔΔCq^ method. *Gapdh* gene was used as a housekeeping gene for mRNA expression. The primers are shown in Table 1.

### 2.9. Immunofluorescence Staining Assay

NSCs were plated into 24-well culture plates with cell climbing slices, and cells were cultured with PTE (30 *μ*g/ml) for 5 days. Cells were washed twice with PBS, fixed in 4% paraformaldehyde for 20 min at room temperature, permeabilized by 0.5% Triton X-100 for 15 min at 37°C, and blocked in 10% goat serum for 30 min at 37°C. The primary antibodies were incubated overnight at 4°C and the secondary antibody was incubated at room temperature for 1 hour. Cells were incubated with 4′,6-diamidino-2-phenylindole (DAPI) for 5 min and mounted by antifade mounting medium. The positive expression of cells was observed by using laser scanning confocal microscopy and the image was taken by ZEN 2 software. The following antibodies were used: rabbit anti-TET1 antibody (1 : 1000, polyclonal, Abcam, Cambridge, UK, no. ab191698), mouse anti-FoxA2 antibody (1 : 500, monoclonal, Abcam, Cambridge, UK, no. ab60721), rabbit anti-tyrosine hydroxylase antibody (1 : 500, polyclonal, Abcam, Cambridge, UK, no. ab112), mouse anti-5-hydroxymethylcytosine (1 : 10000, monoclonal, Active Motif, Carlsbad, USA, no. 39999), DAPI (1 : 1000, Beyotime, Shanghai, CN, no. C1002), goat anti-rabbit IgG H&L (Alexa Fluor 488) (1 : 1000, polyclonal, Abcam, Cambridge, UK, no. ab150077), and goat anti-mouse IgG H&L (Alexa Fluor 647) (1 : 1000, polyclonal, Abcam, Cambridge, UK, no. ab150119).

### 2.10. Coimmunoprecipitation (Co-IP) Analysis

NSCs were plated into 100 × 100 mm cell culture dish and differentiation was induced by PTE (30 *μ*g/ml) for 5 days. Coimmunoprecipitation analysis was performed by using Pierce™ Crosslink Magnetic IP/Co-IP Kit (Thermo) according to the manufacturer's instructions. Briefly, the first is binding of antibody to protein A/G magnetic beads and the final concentration of antibody (mouse anti-FoxA2 antibody, monoclonal, Abcam, Cambridge, UK, no. ab60721) was 5 *μ*g per sample. Then, the bound antibody was crosslinked, and the antibody-crosslinked beads were stored at 4°C. Cell protein was extracted, and the protein concentration was determined. Finally, the protein was added to the antibody-crosslinked beads for immunoprecipitation and the beads and proteins were magnetically separated, and the proteins were detected by western blot analysis.

### 2.11. siRNA Transfection

siRNA silencing of *Tet1* or *Foxa2* was performed. The silencing fragments of *Tet1* and *Foxa2* were designed and synthesized by RIBOBIO Company (Guangzhou, CN.). Single cell suspension was prepared by mechanical dissociation. Cells were plated into 6-well culture plates and cultured with serum-containing medium (10% FBS in DMEM/F12). When the cells were grown to 70%–90%, siRNA was transfected into cells by Lipofectamine® 2000 reagent (Invitrogen) according to the manufacturer's instructions, and the final concentration of transfected siRNA was 50 nM. After siRNA was transfected for 24 h, the expression of *Tet1* and *Foxa2* was assessed by qRT-PCR, and the efficiency of silencing was evaluated; the siRNA fragment with the best silencing efficiency was selected for subsequent experiments.

siRNA was transfected for 24 h, and cells were cultured with PTE (30 *μ*g/ml) for 24 h. The expression of *Th* gene mRNA level was analyzed by using qRT-PCR.

### 2.12. Statistical Analyses

Data in this study were expressed as mean ± standard deviation. Data analysis was done by using GraphPad Prism7 software, at least three independent experiments in each group. The data of in vivo experiment were analyzed using two-way ANOVA and Tukey's test for multiple comparisons; *t*-test (two-tailed) was used for the comparison between two sample groups. The *P* value <0.05 was considered as a significant difference.

## 3. Results

### 3.1. Effect of PT on a Rat Model of PD

**Figure 1:**
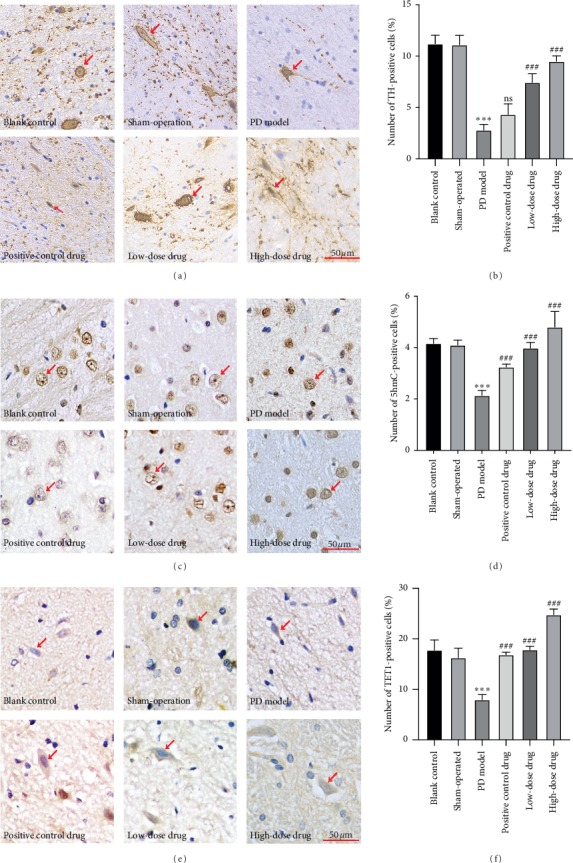
Effects of PT on substantia nigra (SN) of midbrain for PD rats (400x). (a) Staining of TH in rat brain (SN) tissue sections by IHC analysis (magnification: 400x). (b) Statistical analysis of the positive rate of TH-positive cells by using ImageJ software. (c) Staining of 5hmC in rat brain (SN) tissue sections by IHC analysis (magnification: 400x). (d) Statistical analysis of the positive rate of 5hmC-positive cells by using ImageJ software. (e) Staining of TET1 in rat brain (SN) tissue sections by IHC analysis (magnification: 400x). (f) Statistical analysis of the positive rate of TET1-positive cells by using ImageJ software. The positive cells were stained brown, marked by red arrow (↑). ^*∗∗∗*^*P* < 0.01 compared with blank control; ^###^*P* < 0.01 compared with PD model. ns: not significant; *P*: *P* values. The description of the statistical analyses is provided in Supplementary Table S1.

We established a rat model of PD to determine if PT can induce differentiation of NSCs into dopaminergic neurons. We detected TH expression in brain tissue sections and observed TH-positive cells in the model group. The results showed a significant reduction compared with the control group (Figure 1(a)). Simultaneously, we observed a significantly higher number of positive cells in the drug groups compared with the PD model group (Figures 1(a) and 1(b)). To examine the mechanisms of DNA demethylation in the rat PD model, we detected the expression of 5hmC in brain tissue sections. The results showed significant downregulation in the PD model group compared with the control group. Further, both the high-dose drug and low-dose drug groups showed significant upregulation compared with the PD model group (Figures 1(c) and 1(d)). Additionally, we detected expression of TET1, as the rate-limiting enzyme of 5hmC production. We observed that the variable trend of TET1 expression was consistent with 5hmC production. Compared with the control group, TET1 expression was decreased in the PD model group, while the high-dose drug and low-dose drug groups showed a significant increase compared with the PD model group (Figures 1(e) and 1(f)).

### 3.2. Effect of PTE on Differentiation of NSCs

To investigate the mechanism by which PT induces differentiation of NSCs into dopaminergic neurons, NSCs were differentiated ex vivo using PTE.

**Figure 2:**
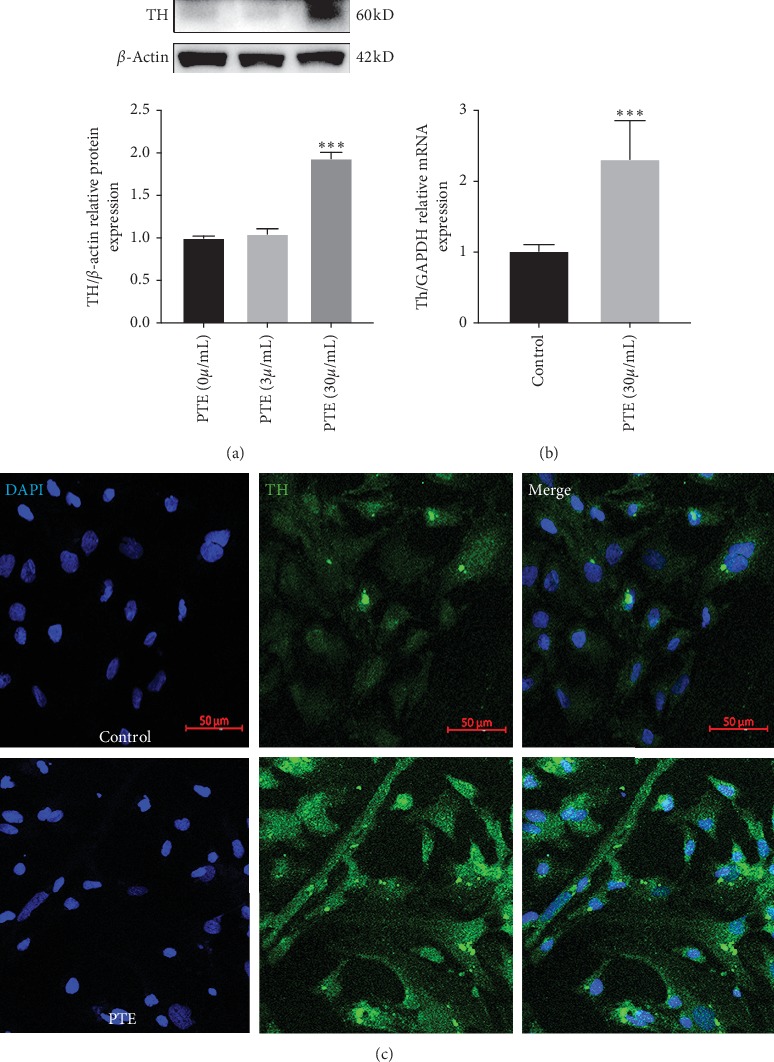
Effects of PTE on TH expression. (a) Western blot analysis for dopaminergic marker tyrosine hydroxylase (TH). ^*∗∗∗*^*P* < 0.01 compared with PTE (0 *μ*g/ml). (b) Expression of mRNA level of Th by quantitative real-time PCR (qRT-PCR) analysis, PTE (30 *μ*g/ml). (c) Immunofluorescence staining of TH expression. Staining is shown for TH (green), with DAPI for nuclear staining (scale bar: 50 *μ*m). NSCs were differentiated into dopaminergic neurons cultures with PTE (30 *μ*g/ml) for 5 days. ^*∗∗∗*^*P* < 0.01 compared with control. *P*: *P* values. The description of the statistical analyses is provided in Supplementary Tables S2 and S3.

NSCs were cultured with serum-containing medium and treated with PTE (0 *μ*g/mL, 3 *μ*g/mL, and 30 *μ*g/mL) for 5 days, and then cell proteins were extracted for western blotting analysis. We detected the expression of TH, which is a dopaminergic biomarker. Our results showed that TH expression was not changed in the 3 *μ*g/mL PTE group compared with the control group (0 *μ*g/mL). Further, TH expression was significantly increased in the 30 *μ*g/mL PTE group (Figure 2(a)). Consequently, we selected a dose of 30 *μ*g/mL for further experiments. Corroboratively, mRNA levels of the *Th* gene were consistent with the trend observed with TH protein levels, with significantly higher *Th* mRNA levels in the PTE group compared with the control group (Figure 2(b)). Immunofluorescence showed that PTE increased the number of TH-positive neurons in differentiated NSCs. In NSC culture not containing PTE, only a few cells were spontaneously differentiated into TH-positive cells. Compared with the control group, TH-positive cells were significantly increased in NSCs cultured with PTE (Figure 2(c)).

### 3.3. Effect of PTE on Global 5hmC Levels and TET1 Expression during NSC Differentiation

**Figure 3:**
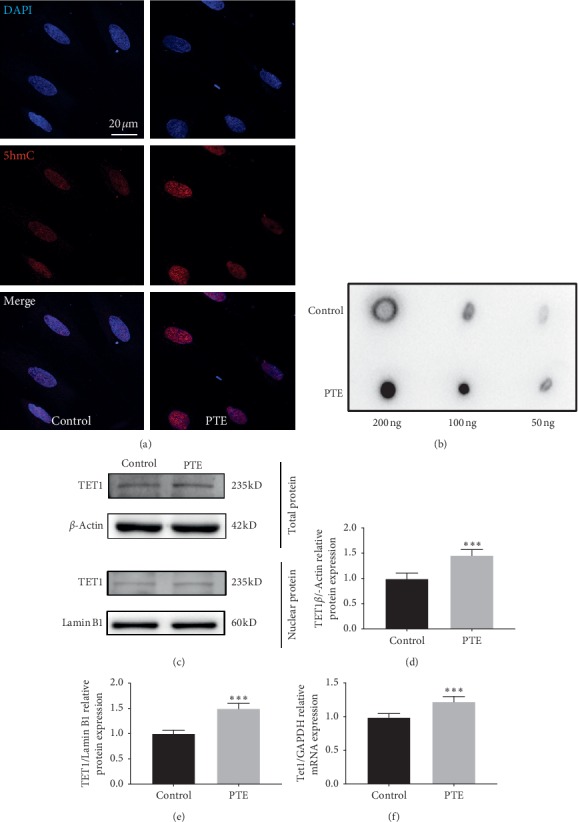
Effect of PTE on global 5hmC levels and TET1 expression. (a) Immunofluorescence staining of 5hmC expression. Staining is shown for 5hmC (red), with DAPI for nuclear staining (scale bar: 20 *μ*m). (b) Dot blot analysis of 5hmC level. (c) Expression of TET1 in total protein and nuclear protein by western blot analysis. (d) Statistical analysis of gray value of TET1 expression in total protein. (e) Statistical analysis of gray value of TET1 expression in nuclear protein. (f) Expression of mRNA levels of Tet1 by qRT-PCR. ^*∗∗∗*^*P* < 0.01 compared with control. *P*: *P* values. The description of the statistical analyses is provided in Supplementary Table S4.

To determine the effect of PTE on DNA demethylation during NSC differentiation, we examined the expression of global 5hmC levels by immunofluorescence (Figure 3(a)). Our results showed that, during NSC differentiation, global 5hmClevels were significantly increased in the PTE group compared with the control group. Simultaneously, we performed a dot blot analysis to detect 5hmC levels (Figure 3(b)). The expression of 5hmC levels was significantly increased in the PTE group compared with the control group.

Next, we examined the expression of TET1 in total protein and nuclear protein extracts by western blot analysis (Figure 3(c)). As expected, TET1 expression in the total protein extract was significantly upregulated in the PTE group compared with the control group (Figure 3(d)). Further, TET1 expression in the nuclear extract was also higher in the PTE group compared with the control group (Figure 3(e)). Additionally, increased *Tet1* mRNA levels were detected in the PTE group (Figure 3(f)). Altogether, these results show that PTE can upregulate the expression of TET1 and 5hmC levels, which may regulate NSC differentiation.

### 3.4. Both PTE and PT Enhancing the FoxA2 Expression

**Figure 4:**
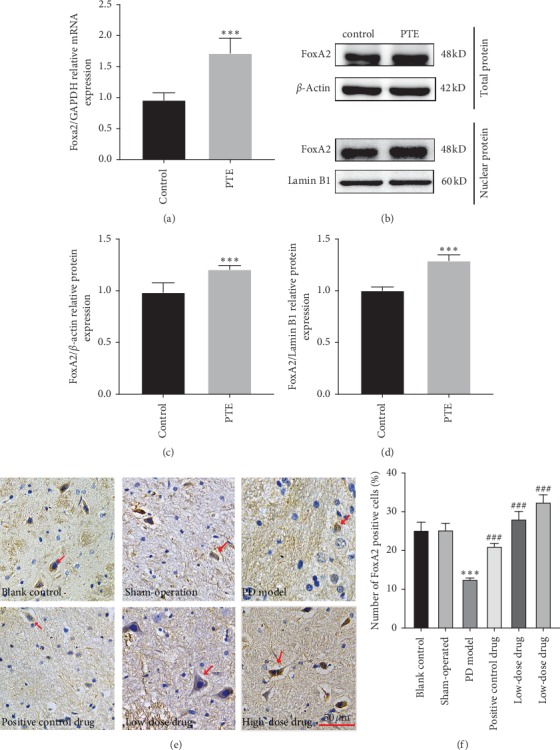
Effect of PTE and PT on FoxA2 expression. (a) Expression of mRNA levels of Foxa2 by qRT-PCR. (b) Expression of FoxA2 in total protein and nuclear protein by western blot analysis. (c) Statistical analysis of gray value of FoxA2 expression in total protein. (d) Statistical analysis of gray value of FoxA2 expression in nuclear protein. ^*∗∗∗*^*P* < 0.01 compared with control. (e) Staining of FoxA2 in rat brain (SN) tissue sections by IHC analysis (magnification: 400x). (f) Statistical analysis of the positive rate of FoxA2-positive cells by using ImageJ software. The positive cells were stained brown, marked by red arrow (↑). ^*∗∗∗*^*P* < 0.01 compared with blank control, ^###^*P* < 0.01 compared with PD model. *P*: *P* values. The description of the statistical analyses is provided in Supplementary Tables S1 and S5.

To investigate which transcription factor is involved in the NSC differentiation induced by PTE, we focused on FoxA2, a critical transcription factor for dopaminergic neurons. Therefore, we examined the effect of PTE on FoxA2 expression during NSC differentiation, ex vivo. The results showed that mRNA levels of *Foxa2* in the PTE group were significantly increased as compared with the control group (Figure 4(a)). Further, the expression of FoxA2 in total protein and nuclear protein was detected by western blot analysis (Figure 4(b)). Results showed that the expression of FoxA2 was significantly increased in the PTE group in total protein and nuclear protein extracts (Figures 4(c) and 4(d)). Moreover, we examined the expression of FoxA2 in brain tissue sections and observed a significant increase in FoxA2 expression in the high-dose drug and low-dose drug groups compared with the PD model group (Figures 4(e) and 4(f)).

### 3.5. Effect of PTE on the Interaction between TET1 and FoxA2

**Figure 5:**
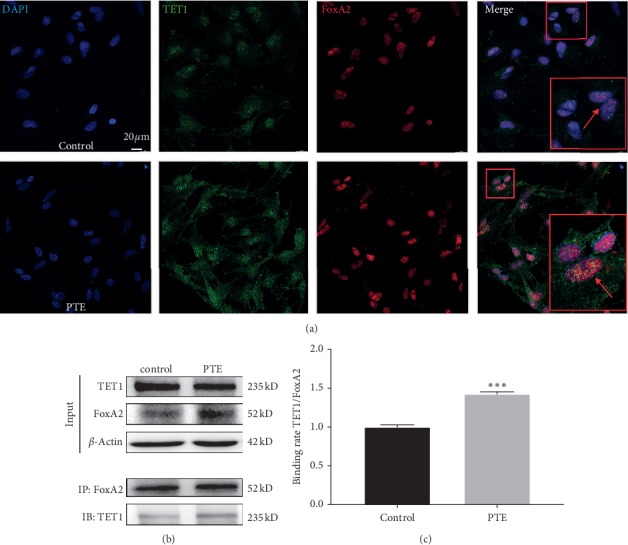
Effect of PTE on the interaction between TET1 and FoxA2. (a) Immunofluorescence colocation staining of TET1 and FoxA2 expression. Staining is shown for TET1 (green) and FoxA2 (red), with DAPI for nuclear staining (scale bar: 20 *μ*m). (b) Coimmunoprecipitation (Co-IP) of TET1 and FoxA2 was assessed by western blot analysis. (c) Statistical analysis of gray value of binding rate between TET1 and FoxA2. ^*∗∗∗*^*P* < 0.01 compared with control. *P*: *P* values. The description of the statistical analyses is provided in Supplementary Table S6.

To investigate the mechanism of PTE on TET1 and FoxA2 during NSC differentiation, we performed immunofluorescence colocalization of TET1 and FoxA2. The results showed that expressions of TET1 and FoxA2 were significantly increased in the nucleus in the PTE group compared with the control group (Figure 5(a)). Subsequently, we performed coimmunoprecipitation (Co-IP) to further examine the relationship between FoxA2 and TET1 protein (Figure 5(b)). Interestingly, we detected an interaction between TET1 protein and FoxA2 during NSC differentiation into dopaminergic neurons. Specifically, an increase in the combination rate of TET1 and FoxA2 was found in the PTE group compared with the control group (Figure 5(c)).

### 3.6. Both TET1 and FoxA2 Are Indispensable for NSC Differentiation into Dopaminergic Neurons

**Figure 6:**
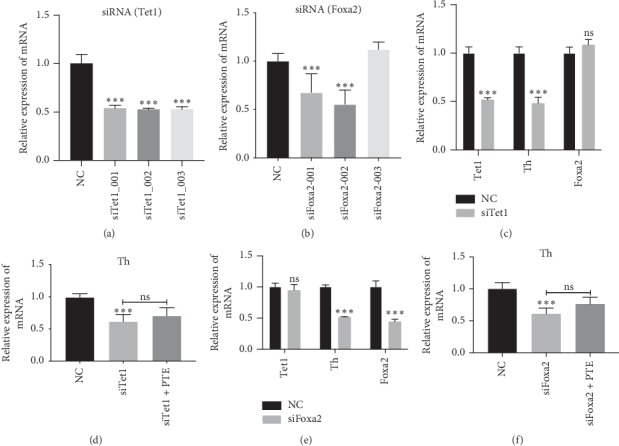
siRNA silencing of Tet1 and Foxa2 gene. (a) siRNA silencing of Tet1 gene. The expression of Tet1 was assessed by qRT-PCR, and the efficiency of silencing was evaluated. (b) siRNA silencing of Foxa2 gene. The expression of Foxa2 was assessed by qRT-PCR. (c) Silencing of Tet1 was done with interference fragment siTet1_001, and the expression of mRNA level of Tet1, Th, and Foxa2 was assessed. (d) Expression of mRNA level of Th by qRT-PCR analysis. siRNA silencing of Tet1 was done in neural stem cells for 24 h and cells were cultured with PTE (30 *μ*g/ml) for 24 h. (e) Silencing of Foxa2 was done with interference fragment siFoxa2_002, and the expression of mRNA level of Tet1, Th, and Foxa2 was assessed. (f) Expression of mRNA level of Th by qRT-PCR analysis. At the differentiation stage, siRNA silencing of Foxa2 were done in neural stem cells for 24 h and cells were cultured with PTE (30 *μ*g/ml) for 24 h. ^*∗∗∗*^*P* < 0.01 compared with control, NC: NControl, ns: not significant; *P*: *P* values. The description of the statistical analyses is provided in Supplementary Tables S7–S12.

Based on our results, we hypothesized that TET1 and FoxA2 are required for the differentiation of NSCs into dopaminergic neurons. Next, we used small interfering RNA (siRNA) to silence *Tet1* or *Foxa2* during the first few days of NSC differentiation. The silencing efficiency was evaluated (Figures 6(a) and 6(b)). Based on the silencing efficiency, we selected *siTet1_001* and *siFoxa2_002* for further experiments. Our subsequent results showed that Foxa2 mRNA levels did not change when *Tet1* was silenced, yet the expression of the *Th* gene was significantly downregulated (Figure 6(c)). Additionally, we found that following the silencing of *Tet1* in the PTE group, expression of *Th* was not significantly different compared with the *siTet1* group (Figure 6(d)). Similarly, after silencing of *Foxa2*, there was no change in *Tet1* expression, while *Th* expression was also significantly decreased (Figure 6(e)). These results show no significant differences in *Th* expression in the PTE group compared with the *siFoxa2* group, after *Foxa2* had silenced (Figure 6(f)).

## 4. Discussion

Currently, treatments for PD are still limited. Clinical treatment of PD is mainly based on drug therapy. Levodopa is considered to be an effective drug for treating PD but can only alleviate the symptoms of patients with PD without achieving a cure. Finding a more effective therapy is an urgent task. Based on the current situation, we are concerned about stem cell therapy, which is considered to be a promising treatment. Moreover, if transplanted stem cells are used to treat PD, complications may arise such as low cell survival rate, poor long-term efficacy, and inability to differentiate directionally. Compared with these caveats, because of the multitarget effects of TCM, this approach has advantages of less toxicity and side effects. Consequently, if PTE can effectively promote NSCs to directionally differentiate into dopaminergic neurons, it will provide more effective and low-cost therapy for PD.

The 6-OHDA-induced rat model of PD causes a significant decrease in the expression of TH, which we used here as a marker to investigate the role of PT. In our study, different outcomes were shown with levodopa or PT treatment. Since levodopa is a raw material that can be used for synthesizing dopamine, its therapeutic mechanism is well known [27]. However, the mechanism of PT is not well known. Nevertheless, because dopamine function could be restored, it is likely that TH expression is upregulated. Hence, we suspect that PT may have an active effect on promoting NSC differentiation into dopaminergic neurons. After PT treatment, the TET1 enzyme and 5hmC results showed an effect on promoting NSC differentiation into dopaminergic neurons, possibly by mediating DNA demethylation. A previous study showed an increase in the percentage of NSC differentiation into dopaminergic neurons, which was closely associated with an increase in 5hmC levels mediated by TET1 [28]. This suggests that TET1 and TET-mediated 5hmC modification are important for the differentiation of NSCs.

To determine the mechanism by which PT can enhance NSCs to differentiate into dopaminergic neurons, we observed the effect of PTE on the differentiation of NSCs ex vivo. Our previous work found that PTE, which is the main active ingredient of PT, could increase the number of TH-positive cells differentiated from NSCs [29]. Moreover, our ex vivo experiments show that PTE can effectively enhance NSC differentiation into dopaminergic neurons and significantly increase the number of dopaminergic neurons, consistent with in vivo experiments.

Our TET1 and 5hmC results suggest that PTE upregulates levels of DNA-5hmC modification, which may regulate NSC differentiation. Another study showed that TET protein interacts with transcriptional factors to regulate cell differentiation [30]. Consequently, we were interested in FoxA2, which is an essential transcriptional factor in the multiple phases of mDA neuronal development. FoxA2 regulates neurogenesis of mDA progenitors and early differentiation of mDA immature neurons, subsequently controlling TH expression in mature mDA neurons during late differentiation [31]. Further, the FoxA2 results suggest that in NSC differentiation into dopaminergic neurons during PTE treatment, FoxA2 may play a critical role. And, the result of in vivo experiment confirms that FoxA2 is increased after treatment with PT. Based on the results, it was suggesting that increasing FoxA2 and TET1 may promote NSCs to differentiate into dopaminergic neurons. Hence, these effects of PTE are likely to be achieved by upregulating FoxA2 and TET1.

However, downregulating FoxA2 may lead to loss of the differentiation potential of ventral mesencephalon-derived neural precursor cells to differentiate into dopaminergic neurons, while activation of endogenous FoxA2 gene by epigenetic regulation may promote dopaminergic neuron production [32]. Furthermore, the positions of FoxA2 binding sites have been identified on the TH promoter, with FoxA2 able to directly activate TH expression in mDA progenitors and mature neurons [33].

In the present study, we show an interplay between TET1 and FoxA2 during NSC differentiation into dopaminergic neurons. PTE enhanced the combination of TET1 and FoxA2, which activated TH expression and promoted NSC differentiation into dopaminergic neurons. Hence, it could be hypothesized that after binding to the TH promoter, FoxA2 guides TET1 to catalyze 5mC to 5hmC, enhancing the transcription of TH and thereby regulating NSC differentiation into dopaminergic neurons.

Finally, we predict that the efficacy of PT for the treatment of PD in vivo may be achieved by promoting endogenous NSCs to directionally differentiate into dopaminergic neurons through regulation of TET1 and FoxA2.

## 5. Conclusions

The present study further clarifies the underlying mechanism by which PTE promotes NSC differentiation. Taken together, PTE has active effects in promoting NSCs to directly differentiate into dopaminergic neurons. Further, PTE increases the efficiency of directional differentiation of NSCs into dopaminergic neurons by increasing the binding rate of TET1 and FoxA2.

## Figures and Tables

**Table 1:** Primers for qRT-PCR.

Gene	Sequence	Supplier
*Gapdh*	Forward: 5′-GTTCAACGGCACAGTCAAGG-3′	Sangon Biotech, Shanghai, CN
	Reverse: 5′-GACGCCAGTAGACTCCACGAC-3′	
*Th*	Forward: 5′-ACTGTGGCTACCGAGAGGAC-3′	
	Reverse: 5′-AATCACGGGCGGACAGTAGA-3′	
*Tet1*	Forward: 5′-CAGGAGAAACGCATGGTACAACAGA-3′	
	Reverse: 5′-CGCTTGCTTGTGTATGGAGTTGG-3′	
*Foxa2*	Forward: 5′-ACGAACTGGCGTTGAAGGAAG-3′	
	Reverse: 5′-CTGAACCTGAGAAGCCTGTGTC-3′	

## Data Availability

All data generated or analyzed during this study are included in this published article. The data used to support the findings of our study are available from the corresponding author upon request.

## Abbreviations

PT: Plastrum Testudinis
PTE: Ethyl acetate extracts from Plastrum Testudinis
NSCs: Neural stem cells
6-OHDA: 6-Hydroxydopamine
TH: Tyrosine hydroxylase
TET1: Ten-eleven translocation 1
FoxA2: Forkhead box A2
5hmC: 5-Hydroxymethylcytosine
5mC: 5-Methylcytosine
TCM: Traditional Chinese medicine
SN: Substantia nigra
mDA: Midbrain dopamine.

## Supplementary Materials

Supplementary Materials In this supplementary material, the statistical analyses in the description of the results have provided the means, standard deviations, and *P* values for each figure caption.
