# Supplementary material for: Plastrum Testudinis Extracts Promote NSC Differentiation into Dopaminergic Neuron by Regulating the Interaction of TET1 and FoxA2
Source: Evid Based Complement Alternat Med. 2020 Apr 20;2020:9104135. doi: 10.1155/2020/9104135 (PMC7189310; doi:10.1155/2020/9104135)
Supplement: Supplementary Materials — In this supplementary material, the statistical analyses in the description of the results have provided the means, standard deviations, and P values for each figure caption. [file 9104135.f1.pdf]

Table1 the description of the statistical analyses for in vivo experiments($\bar{x} \pm s$).

Group	TH	5hmC	TET1	FoxA2
Blank Control group	11.20 ± 0.82	4.17 ± 0.18	17.77 ± 2.00	25.29 ± 2.23
Sham-operation group	11.11 ± 0.90	4.10 ± 0.19	16.26 ± 1.90	25.39 ± 1.85
PD Models group	2.80 ± 0.56***	2.14 ± 0.20***	7.93 ± 1.00***	12.62 ± 0.60***
Positive-drug group	4.33 ± 1.02ns	3.25 ± 0.12###	16.86 ± 0.12###	21.16 ± 0.88###
Low-dose drug group	7.44 ± 0.85###	3.99 ± 0.21###	17.83 ± 0.68###	28.16 ± 2.13###
High-dose drug group	9.50 ± 0.53###	4.81 ± 0.60###	24.80 ± 1.08###	32.56 ± 2.13###

Notes: Blank Control group vs PD Models group:P1, PD Models group vs Positive-drug group: P2, PD Models group vs Low-dose drug group: P3, PD Models group vs High-dose drug group: P4. TH: P1=0.0001, P2=0.0806, P3=0.0001, P4=0.0001. 5hmC: P1=0.0001, P2=0.0002, P3=0.0001,P4=0.0001. TET1: P1=0.0001, P2=0.0001, P3=0.0001, P4=0.0001. FoxA2: P1=0.0001, P2=0.0001, P3=0.0001, P4=0.0001. ***P < 0.01 compared with Blank Control , ###P < 0.01 compared with PD Model. ns: not significant, P: p-values, means±standard deviations: $\bar{x} \pm s$.

Table2 the description of the statistical analyses for Figure2A.

Group	TH($\bar{x} \pm s$)	p-values(compare with PTE(0μg/ml))
PTE(0μg/ml)	1.00 ± 0.02	
PTE(3μg/ml)	1.05 ± 0.06	0.2868
PTE(30μg/ml)	1.93 ± 0.07***	0.0001

Notes: ***P<0.01.

Table3 the description of the statistical analyses for Figure2B.

Group	TH($\bar{x} \pm s$)	p-values(compare with control)
control	1.01 ± 0.09	
PTE(30μg/ml)	2.31 ± 0.55***	0.0002

Notes: ***P<0.01.

Table4 the description of the statistical analyses for Figure3D-F($\bar{x} \pm s$).

Group	TET1(Total protein)	TET1(Nuclear protein)	TET1(mRNA)
control	0.99 ± 0.11	1.00 ± 0.07	0.99 ± 0.06
PTE	1.45 ± 0.12***	1.49 ± 0.10***	1.22 ± 0.08***
p-values(PTE vs control)	0.0001	0.0001	0.0001

Notes: ***P<0.01.

Table5 the description of the statistical analyses for Figure4A,C,D($\bar{x} \pm s$).

Group	FoxA2(Total protein)	FoxA2(Nuclear protein)	FoxA2(mRNA)
control	0.98 ± 0.09	1.00 ± 0.03	0.96 ± 0.12
PTE	1.21 ± 0.04***	1.29 ± 0.06***	1.72 ± 0.24***
p-values(PTE vs control)	0.0003	0.0001	0.0001

Notes: ***P<0.01.

Table6 the description of the statistical analyses for Figure5C($\bar{x} \pm s$).

Group	TET1/FoxA2	p-values(PTE vs control)
control	1.00 ± 0.03	
PTE	1.43 ± 0.03***	0.0001

Notes: ***P<0.01.

Table7 the description of the statistical analyses for Figure6A($\bar{x} \pm s$).

Group	Tet1	p-values(compare with NC)
NC	1.00 ± 0.09	
siTet1_001	0.54 ± 0.03***	0.0012
siTet1_002	0.53 ± 0.01***	0.0009
siTet1_003	0.53 ± 0.03***	0.0010

Notes: ***P<0.01.

Table8 the description of the statistical analyses for Figure6B($\bar{x} \pm s$).

Group	Foxa2	p-values(compare with NC)
NC	1.00 ± 0.08	
siFoxa2-001	0.68 ± 0.19***	0.0002
siFoxa2-002	0.56 ± 0.15***	0.0001
siFoxa2-003	1.12 ± 0.07	0.0088

Notes: ***P<0.01.

Table9 the description of the statistical analyses for Figure6C($\bar{x} \pm s$).

Group	Tet1	Th	Foxa2
NC	1.00 ± 0.06	1.00 ± 0.06	1.00 ± 0.06
siTet1	0.52 ± 0.02***	0.48 ± 0.06***	1.09 ± 0.05 ns
p-values(compare with NC)	0.0002	0.0004	0.1421

Notes: ***P<0.01, ns: not significant.

Table10 the description of the statistical analyses for Figure6D($\bar{x} \pm s$).

Group	Th
NC	0.99 ± 0.06
siTet1	0.62 ± 0.11***
siTet1+PTE	0.70 ± 0.13 ns

Notes: NC vs siTet1(P=0.0001<0.01), siTet1 vs siTet1+PTE(P=0.1388>0.05). ***P<0.01,ns: not significant.

Table11 the description of the statistical analyses for Figure6E($\bar{x} \pm s$).

Group	Tet1	Th	Foxa2
NC	1.00 ± 0.06	1.00 ± 0.03	1.00 ± 0.09
siFoxa2	0.95 ± 0.09 ns	0.52 ± 0.00***	0.45 ± 0.03***
p-values(compare with NC)	0.4405	0.0000	0.0007

Notes: ***P<0.01, ns: not significant.

Table12 the description of the statistical analyses for Figure6F($\bar{x} \pm s$).

Group	Th
NC	0.97 ± 0.04
siFoxa2	0.61 ± 0.09***
siFoxa2+PTE	0.76 ± 0.11 ns

Notes: NC vs siFoxa2(P=0.0037<0.01), siFoxa2 vs siTet1+PTE(P=0.1320>0.05). ***P<0.01,ns: not significant.

Graphical Abstract

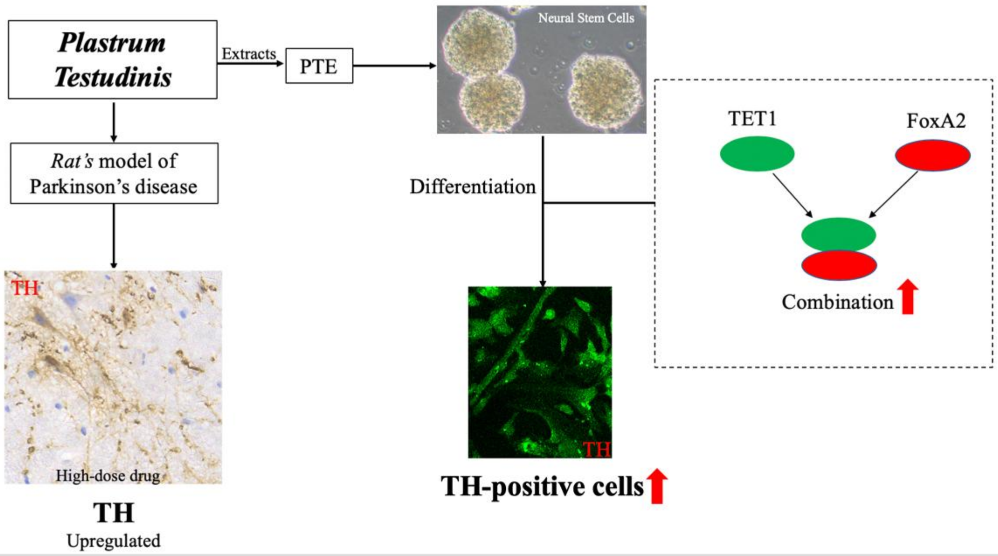

Keywords: Parkinson's disease; Plastrum Testudinis; neural stem cells; TET1; FoxA2
